# Controversy: pulsed field ablation is the standard technique for all the candidates for atrial fibrillation ablation

**DOI:** 10.1093/europace/euaf326

**Published:** 2025-12-24

**Authors:** Luigi Di Biase, Fengwei Zou, Waël Zaher, Jalaj Garg, Serge Boveda, Dhanunjaya Lakkireddy

**Affiliations:** Montefiore Einstein Center for Heart and Vascular Care, Montefiore Medical Center, 111 E 210th street, Bronx, NY 10467, USA; Montefiore Einstein Center for Heart and Vascular Care, Montefiore Medical Center, 111 E 210th street, Bronx, NY 10467, USA; Heart Rhythm Management Centre, Universitair Ziekenhuis Brussel, Heart Rhythm Research Brussels, Postgraduate Program in Cardiac Electrophysiology and Pacing, Vrije Universiteit Brussel, Brussels, Belgium; Heart Rhythm Management Department, Clinique Pasteur, 45 Avenue de Lombez, Toulouse 31076, France; Division of Cardiology, Cardiac Arrhythmia Service, Loma Linda University Health, Loma Linda, CA 92354, USA; Heart Rhythm Management Centre, Universitair Ziekenhuis Brussel, Heart Rhythm Research Brussels, Postgraduate Program in Cardiac Electrophysiology and Pacing, Vrije Universiteit Brussel, Brussels, Belgium; Heart Rhythm Management Department, Clinique Pasteur, 45 Avenue de Lombez, Toulouse 31076, France; Kansas City Heart Rhythm Institute, University of Missouri-Columbia, HCA Midwest Health, 10500, West 105th Street, Suite 200, Overland Park, KS 661215, USA

**Keywords:** Ablation, Atrial fibrillation, Complications, Cryoablation, Pulsed field ablation, Radiofrequency ablation

## Abstract

Pulsed field ablation (PFA) represents one of the most significant technological advances in atrial fibrillation (AF) therapy in recent decades. By harnessing irreversible electroporation, PFA produces myocardial lesions within milliseconds, enabling rapid and efficient pulmonary vein (PV) isolation. Early clinical experience from pivotal investigational device exemption (IDE) trials has shown acute and one-year arrhythmia-free outcomes that are non-inferior to conventional radiofrequency (RF) and cryothermal ablation. The large MANIFEST-17 K registry, encompassing over 17 000 patients treated with the Farawave system, reported an exceptionally low 0.98% major complication rate with no atrio-oesophageal fistula, phrenic nerve injury, or PV stenosis. These findings have accelerated PFA adoption across many centres. However, as experience broadens, nuances in lesion formation and durability are becoming evident. Factors such as contact force, catheter rotation, pulse train configuration, and target tissue geometry influence lesion depth and transmurality. While PV isolation appears consistently durable, data remain limited for non-PV targets such as the posterior wall, mitral isthmus, and cavotricuspid isthmus. Moreover, novel PFA-specific complications including transient left atrial dysfunction, haemolysis, and coronary artery spasm warrant ongoing vigilance. PFA has undoubtedly transformed expectations for procedural safety and efficiency. Yet whether it should already be considered the standard technique for all AF ablation candidates remains an open question. This Controversy piece explores the balance between innovation and evidence, examining whether PFA’s rapid rise represents the inevitable new standard or a technology still undergoing critical refinement.

## Graphical Abstract

**Graphical Abstract:**
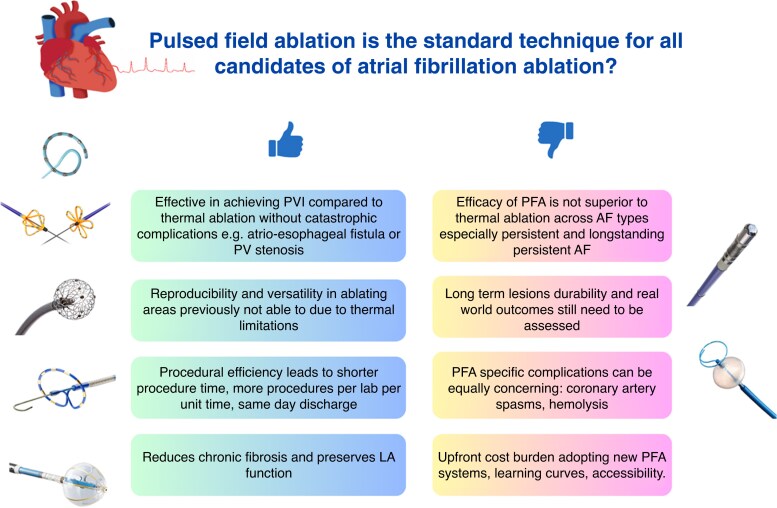

## Figures and Tables

**Table 1:** Safety data from landmark thermal and pulse-field ablation trials

Study/energy	Study design	*N*	Tamponade/pericardial effusion	Stroke /transient ischaemic attack	Atrio-oesophageal fistula	Pulmonary vein stenosis	Persistent phrenic nerve palsy	Coronary spasm	Haemolysis induced renal failure	Procedure-related death
CABANA^16^	RCT	1108 1006 ablated	0.8% (*n* = 8)	0.3% (*n* = 3)	0	0.1% (*n* = 1)	0.1% (*n* = 1)			
FIRE and ICE^17^	RCT	RFA: 376 Cryoablation: 374	RFA: 1.3% (*n* = 5) Cryoablation: 0.3% (*n* = 1)	RFA: 0.5% (*n* = 2) Cryoablation: 0.5% (*n* = 2)	0	0	RFA: 0 Cryoablation: 0.5% (*n* = 2 at 3 months 0.3% (*n* = 1 at >12 months)			No procedure-related deaths
CASTLE-AF^18^	RCT	Thermal: 179	1.7% (*n* = 3)	0	0	0.6% (*n* = 1)				No procedure-related deaths
AATAC^19^	RCT	Thermal: 102	0.98% (*n* = 1)							
ARC-HF^20^	RCT	Thermal: 26 25 ablated	4% (*n* = 1)							0
CAMTAF^21^	RCT	Thermal: 26	3.8% (*n* = 1)	3.8% (*n* = 1)						0
CAMERA-MRI^22^	RCT	Thermal: 33		0						0
TactiFlex PAF IDE trial^23^	IDE	Thermal: 330	0.9% (*n* = 3) Percutaneous: 66.7% (*n* = 2) Surgical: 33.3% (*n* = 1)	0.6% (*n* = 2)	0 1 oesophageal pericardial fistula	0	0			0
SMART-AF^24^	IDE	160 ablated	2.5% (*n* = 4)	0	0	0	0			0
SMART-SF^25^	IDE	159	1.3% (*n* = 2)	0.6% (*n* = 1)	0	0	0			0
Q-FFICIENCY Trial^26^	IDE	166 ablated	1.2% (*n* = 2)	0	0	0	1.2% (*n* = 2)			0
ADVENT^5^ (PFA vs. thermal) (PFA- Boston Scientific Farapulse system Thermal: RFA or cryoablation)	RCT	PFA *n* = 305; thermal *n* = 302	PFA: 0.7% (*n* = 2) Thermal: 0	PFA: 0.3% (*n* = 1) Thermal: 0.3% (*n* = 1)	0	0	PFA: 0 Thermal: 0.7% (*n* = 2)			PFA: 0.3% (*n* = 1) due to perforation/tamponade Thermal: 0
PULSED AF pivotal ^7^ (PFA—Medtronic PulseSelect system)	IDE	300	0.33% (*n* = 1)	0.33% (*n* = 1)	0	0	0			0 procedure-related deaths
MANIFEST-PF survey^27^ (Farawave Farapulse-Boston Scientific system)	Registry	1758	0.97% (*n* = 17) Percutaneous: 0.74% (*n* = 13) Surgical: 0.23% (*n* = 4)	0.39% (*n* = 7)	0	0	0 Transient phrenic injury: 0.46% (*n* = 8)	0.06% (*n* = 1)		0.06% (*n* = 1)
MANIFEST-PF^1^ (Farawave Boston Scientific system)	Registry	1568	1.1% (*n* = 18) Percutaneous: 0.8% (n = 14 Surgical: 0.1% (*n* = 2)	0.4% (*n* = 6)	0	0	0.06% (*n* = 1) Transient phrenic injury: 0.4% (*n* = 6)	0.1% (*n* = 2)	0	0.06% (*n* = 1)
MANIFEST-17K^28^ (Farawave Farapulse-Boston Scientific system)	Registry	17 642	0.36% (*n* = 63) Percutaneous: 0.32% (*n* = 56) Surgical: 0.04% (*n* = 7)	0.12% (*n* = 22)	0	0	0 Transient phrenic injury: 0.06% (*n* = 11)	0.14% (*n* = 25) Proximity-related coronary artery spasm: 88% (*n* = 22) Generalized coronary spasm: 12% (*n* = 3)	0.03% (*n* = 5)	0.03% (*n* = 5)
MANIFEST-US^29^ (Farapulse-Boston Scientific system)	Registry	41 968	0.16% (*n* = 65)	0.10% (*n* = 40)	0	0	0 Transient phrenic injury: 0.01% (*n* = 5)	0.10% (*n* = 40) Proximity-related coronary artery spasm: 75% (30/40): Generalized coronary spasm: 25% (10/40)	0.10% (*n* = 7)	30-day mortality: 0.04% (*n* = 16) 0.019% (*n* = 8) with unexplained sudden death
ADVANTAGE AF^30^ (Farawave Boston Scientific system)	IDE	260	0	0	0	0	0 Transient phrenic injury: 0.4% (*n* = 1)	0.4% (*n* = 1) Presenting as myocardial infarction	0.4% (*n* = 1)	0
Sphere per AF^31^ (Sphere 9, Affera Mapping and Ablation system Medtronic)	RCT	PFA: 212 Thermal: 208	0	0	0	0	0			0
AdmIRE trial^32^ (Variable-loop circular catheter, Varipulse, Biosense Webster)	IDE	272	1.1% (*n* = 3)	1.1% (*n* = 3)	0	0	0			0
inspIRE Study^33^ (Variable-loop circular catheter, Biosense Webster)	IDE	Wave I: 40 Wave II: 186	0	0	0	0	0			0

## Introduction

https://orcid.org/0000-0001-6508-4047Di BiaseLuigi
Montefiore Einstein Center for Heart and Vascular Care, Montefiore Medical Center, Bronx, NY 10467https://orcid.org/0000-0001-6355-8843ZouFengwei
Montefiore Einstein Center for Heart and Vascular Care, Montefiore Medical Center, Bronx, NY 10467

The management of atrial fibrillation (AF) has witnessed a paradigm transformation in the past decade. As therapeutic focus is shifting from rate control to rhythm control, and from a medication-first strategy to consideration of ablation as a first-line option, catheter ablation is now on the forefront of the battle against the AF epidemic. Recent clinical trials and updated AF management guidelines have expanded the indications for early rhythm control and catheter ablation, opening the door for a substantially larger pool of eligible patients.^1–3^

Globally, an estimated 59.7 million people were living with AF in 2019, with ∼4.8 million new cases diagnosed each year.^4^ Yet, only estimated about 600 000 AF ablations are performed annually worldwide.^5^ With the number of yearly performed AF ablation at only 1% of the prevalent AF population and 12% of the annual incidence, the striking mismatch underscores how far we as a field are lagging behind the disease burden. The next major challenge is how to scale access and availability of AF ablation safely and efficiently.

Pulsed field ablation (PFA) represents one of the most exciting technological leaps in AF therapy in decades. Using irreversible electroporation, PFA creates lesions within milliseconds. ‘Single-shot’ pulmonary vein (PV) isolation are reportedly achieved with one brief pulse train, dramatically shortening lesion formation time compared with radiofrequency (RF) or cryothermal ablation in orders of magnitude. Moreover, pre-clinical studies suggest that PFA lesions are tissue-selective and non-thermal, sparing structures such as the phrenic nerves and, importantly, the oesophagus.

Early clinical experience from pivotal investigational device exemption (IDE) trials demonstrated that acute and 1-year freedom from atrial arrhythmias with commercial PFA systems is non-inferior to thermal ablation.^6–11^ The large real-world MANIFEST-17K registry, which evaluated over 17 000 patients treated with the Farawave system, reported an exceptionally low 0.98% major complication rate with no instances of atrio-oesophageal fistula, persistent phrenic nerve injury, or PV stenosis.^12^ Together, these reassuring non-inferiority and safety data and the efficiency of PFA have led many institutions to adopt it as their preferred energy source within a remarkably short timeframe.

### Should it be?

Initial enthusiasm surrounding PFA was fuelled by the promise of rapid, transmural, and durable lesions with minimal collateral injury. However, as clinical experience deepens, the field is beginning to appreciate the nuances of lesion formation—including the importance of contact force, catheter rotation, pulse train configuration, lesion transmurality, inability to assess reversible vs. irreversible electroporation due to acute stunning and many more new safety concerns.^13–15^ Commercial systems rarely deliver a single pulse. Rather, they use multiple pulse trains to enhance lesion durability and consistency as we better understand the boundaries of reversible vs. irreversible electroporation.

While transmural isolation of PVs appears consistently achievable, lesion depth and durability in non-PV regions (e.g. posterior wall, mitral isthmus, cavotricuspid isthmus, and superior vena cava) remain areas of active investigation with early evidence noting limited transmurality and durability.^16–19^ Due to the ease of use, PFA is increasingly being applied beyond PV isolation in redo procedures and atrial flutter circuits, expanding its potential role but also exposing new uncertainties.

Real-world outcomes thus far suggest that efficacy is comparable to conventional thermal energy, without evidence of superiority. Factors such as catheter design, contact stability, and lesion density may determine long-term success as evidence continues to evolve.

The virtual disappearance of devastating thermal complications namely atrio-oesophageal fistula, PV stenosis, and phrenic nerve palsy is one of PFA’s most compelling advantages. Yet, electroporation-specific safety concerns are beginning to surface. Reports from the NEMESI-PFA registry and others describe instances of left atrial mechanical dysfunction, haemolysis, and coronary artery spasm.^19^ One may question, while PFA may mitigate the most feared thermal complications, we must remain vigilant about safety trade-offs from PFA-specific effects.

Needless to say, PFA stands as one of the most exciting and transformative advances in AF ablation since the inception of catheter-based therapy. The technology has rapidly redefined procedural efficiency and safety expectations.

The quest for its optimal application continues as we learn more about this transformative technology. Here, we present a major controversy on how quick and whole-heartedly should we embrace the new energy of choice or will there be a comeback for thermal ablation with ongoing investigation on the role of PFA. Should PFA be the standard for all candidates? How does PFA efficacy differ by AF phenotype (paroxysmal vs. persistent vs. long-standing persistent)? What is the durability at PV and non-PV targets? How is overall comparative safety? What are the implications for scalability and health systems?

This debate will shape the next era of AF management, a critical and exciting moment for electrophysiology.

### Data availability

There are no new data associated with this article.

## Pro

https://orcid.org/0000-0002-1280-7042BovedaSerge
Heart Rhythm Management Department, Clinique Pasteur, 45 Avenue de Lombez, Toulouse 31076, France
Heart Rhythm Management Centre, Universitair Ziekenhuis Brussel, Heart Rhythm Research Brussels, Postgraduate Program in Cardiac Electrophysiology and Pacing, Vrije Universiteit Brussel, Brussels, Belgiumhttps://orcid.org/0000-0002-0537-9262ZaherWaël
Heart Rhythm Management Department, Clinique Pasteur, 45 Avenue de Lombez, Toulouse 31076, France
Heart Rhythm Management Centre, Universitair Ziekenhuis Brussel, Heart Rhythm Research Brussels, Postgraduate Program in Cardiac Electrophysiology and Pacing, Vrije Universiteit Brussel, Brussels, Belgium

Atrial fibrillation (AF) is epidemically prevalent, imposing a growing burden on patients and health systems.^1,2^ Rhythm control via catheter ablation has demonstrated superiority over medical therapy in improving symptoms, reducing hospitalizations, and, in certain populations, improving survival.^3–5^ Therefore, ablation is a Class I LOE A as a first-line option for paroxysmal AF patients since the ESC 2024 Guidelines.^6^ Historically, this therapy has depended entirely on thermal energy sources, especially radiofrequency (RF) and cryoballoon ablation (CRYO). While effective, these methods are limited by unpredictable lesion formation, risk of collateral injury, quite long procedural times, a steep operator learning curve (particularly for RF), and a variable durability of pulmonary vein isolation (PVI).^7–10^ These limitations have constrained broader adoption and have led to uneven outcomes in real-world practice.

In this context, pulsed field ablation (PFA) has emerged as a technology with the potential to overcome many of these limitations.^11^ Indeed, PFA uses ultrashort, high-voltage electric fields to produce irreversible electroporation of cardiomyocyte membranes, causing cell death in a non-thermal manner, while sparing adjacent non-cardiomyocyte tissue such as nerves, oesophagus, smooth muscle, and vessels.^12,13^ Pre-clinical porcine models and human studies have shown that PFA avoids extracardiac lesions that are seen with RF and CRYO techniques.^11,12^ This tissue selectivity-like thus stands as a foundational advantage: it directly addresses many of the most dangerous risks of thermal historical catheter ablation.^13^

Efficient and safe—from the first generation of available equipment, PFA demonstrated that it was equivalent in terms of success to thermal energies, in the three randomized trials available or presented: ADVENT, Single-Shot Champion, and BEAT AF.^14–16^ Concerning observational studies, one of the earliest large-scale real-world cohorts, the EU-PORIA registry, enrolled over 1200 all-comer AF patients (mean age 66 years old, around 60% paroxysmal AF) across multiple European centres, using the pentaspline PFA catheter for PVI.^17^ In that cohort, median procedure time was about 58 min, with 14 min fluoroscopy duration; major complication rate was low (1.7%), and arrhythmia-free survival at one-year was 74%, with paroxysmal AF patients achieving 80% freedom and 66% for persistent AF.^17^ Similarly, the MANIFEST-17K study is a multi-centre, observational registry including more than 17 000 patients who underwent PFA for AF across 106 international centres worldwide, which represents the largest real-world experience with this technology to date.^18^ The results of this study demonstrated very high feasibility and safety, with a major complication rate of only about 0.7%, and no reported cases of oesophageal injury, atrio-oesophageal fistula, or pulmonary vein (PVs) stenosis.^18^ The acute success rate of PVI was nearly 99.9%, confirming the robustness and reproducibility of the technique in everyday clinical practice.^18^ All these outcomes are at least comparable, to cumulative outcomes with RF or CRYO in similar cohorts,^19,20^ and in some aspect better, especially when one accounts for shorter procedure times. Furthermore, during repeat procedures, durability of PVI was high, with 418/584 PVs (72%) remaining isolated in remapped cases, underscoring durable PVI even in real-world practice, especially among operators still on their learning curve.^17,21^

Critics might point to the relative recency of PFA’s adoption and the shorter follow-up compared to decades of RF and CRYO experience. Indeed, many PFA studies have a follow-up of 12 months or less, however, the accumulation of follow-up data is rapid. The EU-PORIA registry, posterior wall (PW) ablation studies, and the persistent AF plus PW isolation study provide mid-term outcomes that are consistent with durability, safety, and efficacy.^17,22–27^ In contrast, thermal techniques’ adverse events, though rare, are serious and sometimes unpredictable; the risk of atrio-oesophageal fistula with thermal ablation remains a concern, with extremely high mortality when it occurs.^2,9,10^ In contrast, PFA has to date shown near-zero occurrence of this dramatic complication.^14,17,18^

Interestingly, safety advantages of PFA are not limited to rare catastrophic events. Compared to thermal methods, PFA has currently shown greatly reduced rates of collateral injury in more common manifestations: less oesophageal heating, less risk of thermal injury to the phrenic nerve, no chronic PVs stenosis, and less risk of microvascular or vascular injury.^17,18^ These translate into fewer severe as well as minor complications, shorter hospital stay, and fewer unplanned readmissions.^14^ For many patients, especially those with comorbidities or older age, the margin of safety is particularly important in shared decision making.^28^

Efficacy encompasses both acute procedural success and long-term freedom from arrhythmia. Acute PVI with PFA tends to be very high: in many studies >95%.^11,14,15^ Lesion durability, judged by remapping, is strong, even in persistent and redo settings.^21^ One-year arrhythmia-free survival rates for PFA are in the range of 70–80% for paroxysmal AF and somewhat lower but still competitive (60 to 70%) for persistent AF, depending on the lesion set employed.^22,23,26,27^ While longer-term follow-up (beyond 2–3 years) remains limited, early data suggest that the integrity of lesions holds up, and recurrence rates are largely driven by non-pulmonary vein substrates or gaps, not by inherent failure of the PFA lesion modality itself.^29^ Nonetheless, current evidence is largely based on single-arm registries and clinical trials with relatively short follow-up, and uncertainties remain regarding long-term outcomes, further highlighting the need for more robust, longer-term randomized trials to validate these observations.

Versatile—Beyond PVI, PFA has been applied to more extensive lesion sets.^22^ A recent prospective series investigated PW ablation using PFA with pentaspline catheters in 215 patients (about two-thirds undergoing redo procedures). PW ablation was achieved in 100% of cases, with low major complication rates (0.9%), and in redos the PW was found durable in 85% of patients.^30^ Although one-year arrhythmia-free survival in that study was lower (53%) for the PW strategy, likely reflecting a more advanced substrate or redo setting, the demonstration of procedural safety and lesion durability in a more challenging population is promising and suggests that PFA can be safely applied beyond simple PVI, when a substrate ablation strategy has been decided.^22–25^ More recently, in a large prospective study of persistent AF using a strategy of PVI plus PWI delivered via PFA, favourable safety and effectiveness outcomes were reported, further expanding the envelope of what PFA may standardly accomplish.^26,27^ In contrast to the high durability of PVI and PWI, block of mitral isthmus lines and left atrial appendage or coronary sinus isolation is often lost at 3-month remapping, underscoring the limited durability of some extra-PV targeted area, with the pentaspline catheter.^31^ These limitations may be more related to catheter morphology—originally designed for PVI—and line orientation than to PFA energy *per se*, and newer focal catheters are being evaluated to enable more tailored and potentially more durable linear ablation beyond the PVs, although definitive comparative data are not yet available.

Scalability across patient populations is essential if PFA is to be the standard for ‘all candidates’. The data support its use not only in paroxysmal but persistent AF, and also for redo procedures, in older patients, in those with prior ablations, across different centres with variable operator experience. In persistent AF, substrate ablation like PWI and more extensive lesion sets are being explored, and early experience suggests they can be delivered safely and effectively with PFA, but incremental clinical benefit over PVI alone has not yet been demonstrated and remains to be established in dedicated trials.^24–27,30,32^

Preservation of left atrium (LA) function—Unlike thermal energy, which causes coagulative necrosis, inflammation, and fibrotic scarring, PFA is non-thermal and mainly promotes apoptosis while preserving the extracellular matrix, microvasculature, and nerves, thereby limiting chronic fibrosis.^33^

Pre-clinical data demonstrate tissue selectivity with sparing of collateral structures and minimal inflammatory response^34^; clinically, cardiac magnetic resonance shows large, homogeneous acute late gadolinium enhancement (LGE) with modest oedema after PFA that largely resolves by 3 months, in contrast to the persistent LGE seen after thermal ablation.^35^ Atrial wall compliance and LA reservoir/booster function decline acutely after both energy sources but recover by 3 months only after PFA, consistent with reparative healing with minimal chronic fibrosis.^35^ Minimizing fibrotic remodelling is critical to preserving LA function following ablation and could explain the observed improvements in LA strain reservoir,^36^ as well as in LA volume index and left ventricular ejection fraction compared to CRYO,^37^ suggesting that PFA may induce better LA reverse remodelling.

These properties are particularly relevant when ablation extends beyond the PVs in persistent AF: extensive thermal ablation, by inducing extensive fibrotic scarring, is associated with ‘Stiff LA syndrome’, characterized by reduced LA compliance and reduced LA volume.^38–41^ By contrast, emerging comparative data suggest that PFA beyond PVs, preserves LA compliance as well as LA volume,^42–44^ supporting better preservation of LA mechanics by preventing restrictive feature. As such, PFA may maintain LA function, potentially attributed to reduced post-ablative fibrogenesis compared to thermal energy; this should be considered in extensive ablation scenario given the high association of persistent AF with heart failure and the importance of LA function in this population.

Procedural efficiency—Procedural efficiency is another dimension where PFA can offer advantages over thermal energy sources, although the magnitude of this benefit depends on patient anatomy, operator experience and learning curve, sedation strategy and overall lab workflow. Many PFA studies report skin-to-skin procedure times of under one hour for PVI, with fluoroscopy times likewise brief.^17–20^ By contrast, RF point-by-point ablation is more meticulous, and it frequently requires mapping time, contact force optimization, repeated energy applications, and sometimes multiple passes per vein, which cumulatively lead to longer procedures.^14,20^ Shorter procedures may reduce patient risk, improve comfort, reduce resource utilization. At the organizational level, they can also permit more procedures per lab per unit time and facilitate same-day discharge, thereby increasing throughput and reducing waiting lists in health systems, but these are system-level consequences rather than direct clinical benefits.^45,46^

Reproducibility—Operator dependency has also been a long-standing concern in AF ablation. RF ablation requires not only technical skills but high degrees of consistency in catheter stability, contact force, lesion overlap, mapping accuracy. CRYO simplifies certain aspects, and has proven to be more reproducible but it retains risks (e.g. phrenic nerve palsy, inadequate cooling or freezing in certain anatomies).^47^ PFA promises more uniform application: devices such as pentaspline, single-shot balloon or spiral array catheters reduce procedural variability. Early adopter and registry data from EU-PORIA and other studies show that even operators new to PFA achieve favourable outcomes, and that paroxysmal vs. persistent AF outcomes diverge as expected, but not dramatically beyond what thermal techniques show. In EU-PORIA, for example, procedure times did not significantly vary with operator experience, suggesting a relatively shallow learning curve.^17^ Reproducibility across centres and operators is thus expected to be high.

Patient-centred considerations also favour PFA—Reduced procedure time, low of major and minor complications, shorter hospital stays, fewer readmissions, all contribute to improved patient experience.^17,18^ For many patients, the trade-off between procedural risk and symptom relief is heavily influenced by fear of complications, especially life-threatening ones and in an era of shared decision making, patients’ values and preferences regarding safety and recovery time are central to treatment choice.^6^ In this context, it is increasingly common for patients, having gathered their information, particularly online, to request PFA ablation as a first option, because, in their view, PFA is at least as safe as thermal ablation with respect to complications that are largely non-energy-related (e.g. pericardial effusion, vascular access complications, stroke), while the absence to date of dramatic complications (e.g. atrio-oesophageal fistula and symptomatic pulmonary vein stenosis) in large PFA series further reinforces their perception of PFA as the preferred option.

Economic implications must also be considered—While new devices often come with higher upfront costs, PFA’s advantages, as shorter learning curve and shorter procedures, which optimize the use of the EP-lab and improve the workflow, with more patients treated, suggest that overall cost may be lower or at least competitive when considered over a longer horizon.^45,46,48^ Real-world nationwide registry data show low rates of major complication and high procedural efficiency;^49^ these translate into fewer resource demands post-procedure as well. From a health-economic standpoint, the main cost drivers are procedure duration, length of stay, and the need for re-interventions due to complications or reconnection; on which PFA may be advantageous. Health systems under budget constraint will value technologies that reduce downstream costs, even if device acquisition requests an initial investment, however, robust cost-effectiveness analyses of PFA vs. thermal ablation are still limited and their results are likely to differ between healthcare systems.

The story is just beginning—Thermal energies, particularly RF, are >20 years old, with very significant improvements and evolutions since the first-generation catheters. Looking forward, PFA aligns well with emerging techniques and technologies in electrophysiology. Advanced three-dimensional mapping, imaging integration, and artificial intelligence guidance are increasingly important. Because PFA lesions are more binary and less dependent on operator variables, such as contact force or thermal dynamics, they are more amenable to integration with imaging and mapping for real-time lesion assessment. Device design is also currently evolving: balloon, single-shot spiral or spherical arrays, variable-loop biphasic, further increasing applicability and adaptability.^50,51^ This suggests that PFA is not simply a temporary improvement, but foundational technology for upcoming generations of AF therapy, which will also likely contribute to further improving the efficiency, safety, and reproducibility of the procedure.^29,52^

Obviously, no technology is perfect, and PFA also has its limitations. Long-term data beyond 2–3 years are still being collected; there is less experience in persistent or long-standing persistent AF and for ablation in complex substrates (e.g. atrial fibrosis, dilated LA). The optimal energy settings, lesion delivery strategies for non-PV substrates, and long-term follow-up especially in structurally diseased atria are still currently subjects of investigation, and we still have much to learn in these areas. Also, device cost and reimbursement issues may initially slow uptake in some health systems.^45,46^ Rare events such as coronary vasospasm have also been reported, particularly when PFA has been delivered in certain locations like the cavotricuspid or mitral isthmuses and require vigilance.^53^ Haemolysis has likewise been reported, but the incidence appears low—mostly when a very high number of applications are performed—and it rarely has clinical implications, as AKI appears extremely rare when PFA is used in a standardized fashion.^18^ Nevertheless, none of these limitations appear currently to undermine the central acknowledged advantages of safety, durability, efficiency, reproducibility, and patient acceptability.

In sum, the balance of evidence indicates that PFA has progressed without a doubt far beyond proof-of-concept. It delivers high acute success rates, durable lesion formation, favourable clinical outcomes, and an improved safety profile combined with operational efficiencies. For all candidates for AF ablation, paroxysmal, persistent, redo, or first ablation, PFA offers a superior or at least non-inferior therapeutic option compared to RF or CRYO, with fewer downsides. The case is strong that PFA should be considered the standard technique for all AF ablation candidates, not just a premium or experimental alternative.

## Con

https://orcid.org/0000-0002-6857-7418GargJalaj
Division of Cardiology, Cardiac Arrhythmia Service, Loma Linda University Health, Loma Linda, CA 92354, USAhttps://orcid.org/0000-0002-1492-2693LakkireddyDhanunjaya
Kansas City Heart Rhythm Institute, Overland Park, KS 661215, USA

Pulsed field ablation (PFA) has rapidly emerged as an innovative non-thermal energy source for atrial fibrillation (AF) ablation. Its mechanism of irreversible electroporation promises myocardial-specific injury while ostensibly sparing surrounding structures, fuelling enthusiasm that it could supplant radiofrequency ablation (RFA) and cryoablation as the new standard of care. However, despite promising early results, a critical appraisal of the evidence reveals that PFA is not yet a panacea for all AF patients. Variations in efficacy across AF subtypes, questions about lesion durability, novel safety concerns, and practical limitations (cost, training, and access) temper the initial optimism. This second part of the debate examines the current data on PFA—opposing the position that PFA should be the uniform first-line ablation technique for all AF patients—and highlights where further study or caution is warranted.

### Efficacy across AF subtypes: paroxysmal vs. persistent AF

Clinical experience suggests PFA’s effectiveness may depend on AF subtype. In paroxysmal AF, PFA achieves acute pulmonary vein isolation (PVI) rates ∼99% and 12-month freedom from atrial arrhythmias around 75–85%, which is comparable to established thermal approaches.^1,2^ For instance, the *MANIFEST-PF registry* of first-time ablations reported ∼78% one-year freedom from AF/atrial tachycardia in paroxysmal AF patients.^1^ Similarly, a propensity-matched single-centre study found 85% freedom from arrhythmia at 1 year with PFA, compared to 73.8% with RFA and 66.2% with cryoballoon, in patients with paroxysmal AF (differences not statistically significant).^3^ The *BEAT-PAROX-AF* trial, a multi-centre randomized study comparing PFA with optimized contact-force RFA using the CLOSE protocol, reported 12-month single-procedure success rates of 77.2% with PFA and 77.6% with RFA (adjusted difference +0.9%; 95% CI −8.2 to +10.1; *P* = 0.84), indicating that PFA is not superior to state-of-the-art thermal ablation with respect to arrhythmia recurrence.^4^ While acute PVI success was near-universal in both arms, the similar 1-year outcomes suggest that chronic reconnection rates after PFA may still limit durability, especially considering that the RFA comparator used high-quality CLOSE lesions with proven durability in prior studies. These data indicate that for paroxysmal AF, PFA can achieve at least equivalent efficacy to RFA or cryoablation, consistent with *ADVENT* trial, which showed ∼73% arrhythmia-free survival with PFA vs. ∼71% with conventional ablation.^5^

However, the efficacy of PFA in patients with persistent and long-standing persistent AF remains less well established. In the *MANIFEST-PF registry*, 12-month freedom from atrial arrhythmias in patients with persistent AF was ∼71%,^1^ slightly higher than seen with thermal ablation in this harder-to-treat group. Among the small subset of patients with long-standing persistent AF (∼3% of the cohort), 12-month freedom from atrial arrhythmias was ∼73%; however, the limited sample size precludes definitive conclusions.^1^ On the contrary, early real-world series have shown one-year AF free survival as low as ∼66–67% for persistent and long-standing persistent AF (Boston Scientific Farapulse system),^6^ whereas investigational study—*PULSED-AF* (Medtronic PulseSelect system)^7^ has demonstrated one-year efficacy as low as ∼55% in persistent AF. This variability suggests an evolving learning curve and the need for adjunctive lesion sets. Indeed, in *MANIFEST-PF registry*, only ∼11% of patients received posterior wall ablation beyond PVI, and few had linear lesions or other substrate modification.^1^ While persistent AF ablation often requires ablation beyond PVI, early experience shows that using PFA for linear lesions (e.g. roof or mitral isthmus lines) or for coronary sinus or left atrial appendage isolation results in poor long-term lesion durability, highlighting important limitations of current PFA systems for non-PV targets.^8,9^ These areas may still require the conventional RF energy for a more durable lesion. Taken together, emerging data suggest that PFA’s incremental efficacy is largely confined to paroxysmal AF, whereas studies in non-paroxysmal AF have not shown meaningful incremental benefit. Until more definitive data emerge, positioning PFA as the default modality for complex AF substrates is unwarranted.

### Long-term lesion durability

A fundamental requirement for any ablation technology to become standard is durable ablation lesion. Unlike thermal injury, which causes necrosis and scarring, the longer-term tissue response to electroporation is an area of active investigation. Recent studies raise important concerns regarding the durability of PVI: in the multi-centre *EU-PORIA* registry, durable PVI (i.e. all PVs in an individual patient) was found in only 38% patients.^10^ Notably, durability was lower in patients with persistent AF (only ∼66% of veins remained isolated) compared to ∼76% in paroxysmal AF.^10^ Gaps and PV reconnections continue to occur with PFA, likely reflecting suboptimal energy delivery or oedema that resolves over time. The link between durable PVI and clinical success was highlighted by Turagam et al (in *MANIFEST-PF registry*), who reported that centres achieving >50% durable PVI had significantly higher 1-year success rates (81% vs. 71%, *P* < 0.001) than centres with less durable lesions.^1^ This implies PFA outcomes will depend heavily on improving lesion durability through technique and technological advances. Early histopathology data have shown PFA lesions can be transmural and fibrotic by 1–2 months post-ablation,^11,12^ suggesting that with correct energy dosing, permanent conduction block is achievable. If an ultra-rapid PFA procedure leads to higher redo rates, the initial efficiency gains are nullified. Therefore, until PFA’s long-term lesion durability (for PV and non-PV lesions alike) is demonstrated at scale, a cautious stance is warranted.

### Safety profile: trading one set of risks for another

Despite three decades of refinement in RFA, including advances in procedural techniques, adjunctive tools, and workflow optimization, collateral injury—while significantly reduced—has not been entirely eliminated. In contrast, the primary safety advantage of PFA lies in its non-thermal mechanism of action, which substantially mitigates the risk of catastrophic complications. Clinical experience to date indicates that PFA effectively avoids adverse events such as PV stenosis, oesophageal injury or fistula, and phrenic nerve palsy. In *MANIFEST-PF registry*, *zero* PV stenoses or atrio-oesophageal fistulas were reported.^1^ Even phrenic nerve injury—a well-known complication of thermal ablation—was markedly reduced; only one case of transient phrenic palsy (0.06%) was seen.^1^ This safety signal was also demonstrated in the largest U.S. dataset to date: the *MANIFEST-US registry* of 41 968 patients—*zero* cases of atrio-oesophageal fistula, *zero* PV stenosis, and *zero* persistent phrenic nerve palsy, confirming that these catastrophic thermal ablation complications have not emerged even at scale. This favourable safety profile is attributed to the tissue-selective nature of electroporation, which preferentially ablates cardiomyocytes while largely preserving collagen-rich structures such as vascular walls, neural tissue, and the oesophagus.^13–15^

While, these significant benefits could justify PFA as first-line for AF ablation, recent data reveal that PFA is not free of adverse events, and its risk profile differs from thermal ablation in ways that are only beginning to be understood (*Table 1*). The *NEMESIS*-*PFA* study, a multi-centre registry, demonstrated a significantly greater release of biomarkers of tissue and blood injury—higher peak cardiac troponin (median ∼13 551 vs. 128 ng/L), lactate dehydrogenase, and plasma-free haemoglobin, as well as greater drops in haemoglobin levels with PFA as compared to RFA. Post-PFA patients also had a larger acute decrease in left atrial ejection fraction (∼20% vs. 5% after RFA).^34^ The *MANIFEST-US registry* identified haemolysis-associated acute renal failure requiring dialysis in 0.02% of patients (*n* = 7). Although rare, all cases occurred immediately post-procedure and were linked to high application counts or underlying renal vulnerability. Three of seven cases were fatal due to concurrent complications (stroke, tamponade, cardiogenic shock). Together, these findings suggest PFA causes more widespread atrial muscle stunning and intra-procedural hemolysis.^34^ In other words, PFA avoids thermal injuries at the cost of increased myocardial and blood cell injury—the long-term significance of which remains unclear.

Other emerging safety concerns include:

- Silent cerebral emboli: Silent cerebral emboli (SCE) and intra-procedural microbubbles are a non-trivial safety signal with PFA and merit deliberate monitoring. Biophysically, high-voltage pulses can generate gas at the electrode–blood interface through electrolysis and rapid degassing/cavitation, producing micro bubbles or macro bubbles from coalescence, leading to cerebral and other systemic embolizationmechanistically distinct from heat-driven bubble formation seen with RFA. Experimental and *in vitro* work confirms pulse-dependent gas liberation and shows that waveform engineering (e.g. biphasic and shorter pulses) reduces bubble formation. In addition, the risk of SCE varies substantially among different PFA catheter systems and is not a hallmark of the energy source itself.^35^
  In *ADVENT* sub-study, SCEs were detected in a small minority of PFA patients (3 SCEs among 34 PFA patients, none in 37 RFA patients), with one transient ischaemic attack (PFA arm) and one overt stroke (RFA arm) (neither in the MRI-imaged cohort).^36^ In a recently published meta-analysis pooling 10 456 patients, estimated SCE rates were ∼14.4% for PFA vs. ∼17.7% for RFA and ∼20.8% for cryoablation (no significant difference between groups).^37^ Although most lesions are clinically silent, registry-level stroke rates after PFA remain very low (∼0.1–0.3%),^28,38^ and overt cerebrovascular events have been linked to modifiable factors such as catheter exchanges, sheath/air management, and sub-therapeutic activated clotting time (ACT).
  Pulse waveform parameters are also important: using biphasic (alternating polarity) pulse trains and minimizing total charge lowers electrolysis. Notably, anodal irreversible electroporation pulses produce fewer bubbles than cathodal ones, and sub-RF alternating-current pulses avoid electrolysis altogether.^39^ In summary, PFA microbubbles likely reflect gas liberated by pulse-induced electrolysis, heating, and degassing, and—while they can cause MRI-visible ‘silent’ lesions— and therefore, rigorous de-airing and continuous flushing, un-interrupted anti-coagulation with ACT > 300 s, attention to pulse-delivery protocols, and consideration of post-procedure neurological assessment or MRI in higher-risk cases is essential.
- Coronary vasospasm and arterial injury: Coronary artery vasospasm has emerged as a rare but recognized complication of PFA, particularly when lesions are delivered adjacent to epicardial vessels. In large clinical registries, the overall incidence of coronary spasm during PFA remains low—for example, the *MANIFEST-17K* study (*n* = 17 642) reported spasm in only 25 patients (0.14%),^28^ most of which were ‘proximity-related’ to coronary arteries. By comparison, historical data from thermal ablation show similarly low coronary spasm rates (e.g. ∼0.04% with RFA and ∼0.34% with cryoablation).^14^  *MANIFEST-US* registry provides the largest real-world estimate: coronary spasm occurred in 0.10% of all patients, with 75% being proximity-related [typically cavotricuspid isthmus (CTI) or mitral isthmus ablation] and ∼25% representing generalized spasm. Proximity-related spasm occurred in 30 patients (0.07%), usually intra-procedural (most often during CTI ablation) and marked by transient ST-elevation. Generalized coronary spasm occurred in 10 patients (0.024%). Overall, significant clinical sequelae were observed in 16.6% (*n* = 5) cases with proximity-related spasm [Ventricular fibrillation (VF) or ST-elevation and air embolism, hemodynamic instability or AV block], and 70% (7 out of 10 cases) with generalized coronary spasm cases (AV block, sinus arrest, VF arrest, cardiogenic shock). Studies have shown that PFA delivered at high-risk sites (e.g. the mitral or CTI) frequently provokes spasm. For instance, one study found angiographic left-circumflex spasm in 7 of 17 patients (41%) during PFA of the mitral isthmus (vs. 0% with RFA),^40^ and Reddy et al. reported severe right coronary spasm in 5/5 patients during PFA of CTI unless nitroglycerin was administered.^41^ The mechanism of PFA-induced spasm is not fully understood but is thought to involve direct pulsed-electric-field effects on vascular smooth muscle (causing transient contraction) and possibly reflex autonomic influences. Pre-clinical studies have demonstrated that PFA across a coronary vasculature induced acute vasoconstriction in ∼45–49% of ablations,^42^ and chronic neointimal hyperplasia and mild stenosis in most vessels (≈87.5%) exposed to epicardial PFA.^43^ In humans, a study by Tam *et al.* observed severe coronary spasm (>70% constriction) in 45% of ablations near the right coronary artery or left circumflex despite nitroglycerin prophylaxis.^44^ Over 3 months, treated vessels showed mild vessel wall thickening and a ∼10% reduction in luminal area on OCT imaging—all remained asymptomatic or required any interventions at follow-up.
  Although PFA reduces the risks associated with thermal injury, it may acutely provoke coronary vasospasm, particularly when delivered near epicardial vessels. To minimize this risk, procedural strategies such as prophylactic administration of high-dose nitrates,^41^ avoidance of ablation near arterial hot spots,^40^ and/or deactivation of electrodes facing the coronary vasculature could be considered. Therefore, careful case planning, vigilant ECG/angiographic monitoring, and/or routine use of nitrates/vasodilators should be considered to reduce the risk of clinically significant vasospasm.
- Cardiac perforation/tamponade: While rates are low, PFA has not eliminated mechanical complications. In the *MANIFEST-17K* registry, the incidence of tamponade was 0.36%, with nearly 90% successfully managed by percutaneous drainage.^28^ Similarly, the *MANIFEST-PF survey* (*n* = 1758) reported a slightly higher early experience rate of 0.97%,^27^ which declined as operator familiarity increased—highlighting a procedural learning curve. In the randomized *ADVENT* trial, tamponade occurred in 0.7% of PFA patients and in none of those treated with thermal ablation, though the overall event rate was too low to establish statistical differences between modalities.^5^ In the *MANIFEST*-*US*, the incidence of tamponade was 0.16% of 41 968 cases (65 patients), with 77% managed percutaneously and 23% surgically. This rate is lower than early European experience (0.36% in MANIFEST-17K) and ADVENT trial suggesting a learning-curve effect. These rates compare favourably with those reported for conventional thermal ablation cohorts (0.5–1.0% cases).^45^
  Importantly, mechanistic analyses suggest that most PFA related tamponades are mechanical rather than energy-mediated. Perforations are often attributed to transseptal puncture, guidewire or sheath trauma, or aggressive catheter manipulation, whereas energy-related perforation (e.g. steam pops with RFA) is virtually absent with PFA. Small pericardial effusions without hemodynamic compromise are also occasionally seen, likely reflecting inflammatory pericarditis or microvascular leakage rather than frank perforation.
  Mortality: In the *MANIFEST-US registry*, the 30-day mortality rate was 0.04% (16 of 41 968 cases), with half attributable to identifiable causes such as stroke, tamponade, retroperitoneal bleeding or sepsis or cardiogenic shock related to renal failure, and the remainder representing unexplained sudden deaths or cardiac arrests (0.019%) occurring between 2 and 18 days post-ablation. This pattern is consistent with MAUDE database analyses, where early sudden deaths constituted the majority of unexplained fatalities. While these rates are low, the proportion of unexplained early deaths is notable and warrants further investigation—particularly given that large registries of thermal ablation have not reported a comparable burden of early unexplained mortality. Whether this reflects unique electrophysiologic or autonomic effects of PFA, system-related factors, or post-procedural care variability remains undetermined.

In summary, while PFA demonstrates clear safety advantages in select domains—most notably the apparent elimination of atrio-oesophageal fistula, pulmonary vein stenosis, and sustained phrenic nerve injury—it also introduces a distinct spectrum of complications (*Table 1*). The *MANIFEST-US* registry highlights this duality, reporting low but non-trivial rates of coronary vasospasm (0.10%), tamponade (0.16%), dialysis-requiring acute kidney injury (0.02%), stroke (0.10%), and early unexplained sudden death (0.019%). Although the absence of thermal injury represents meaningful progress, emerging concerns—such as elevated myocardial biomarkers, atrial stunning, and haemolysis—raise questions about the physiologic impact of widespread electroporation. Whether these acute effects carry long-term clinical consequences remains unclear. Thus, while PFA may offer a favourable safety profile in some aspects, its adoption as a default modality requires a balanced understanding of these novel risks.

### Biophysical and mechanistic concerns

Several mechanistic considerations remain unresolved before PFA can be broadly endorsed as a universal approach, with its impact on atrial compliance and mechanical function being of particular interest. Extensive left atrial ablation with thermal energy—particularly RF—has been associated, albeit infrequently, with stiff left atrium (LA) syndrome, characterized by iatrogenic diastolic dysfunction, pulmonary hypertension, and heart failure symptoms. Whether PFA mitigates this risk remains uncertain. Pre-clinical and early clinical evidence suggests that PFA may induce less fibrotic remodelling due to its non-thermal mechanism, potentially preserving atrial compliance.^46^ In fact, studies have demonstrated improvement in LA reservoir, conduit, and contractile strain post PFA ablation—suggestive of enhanced LA performance (independent of AF subtypes), unlike post RFA.^47,48^ On the contrary, recent prospective data indicate that when PFA is extended beyond PVI (e.g. to posterior wall or linear lesions), subclinical impairments in LA compliance may occur. Patients receiving such lesion sets demonstrated larger LA volumes, reduced reservoir strain, and elevated stiffness indices at 4–6 months post-ablation—though none developed clinically overt stiff LA syndrome or pulmonary hypertension, and quality-of-life scores remained comparable.^49^ These findings suggest that atrial stiffening is not exclusive to thermal injury and may occur with any extensive lesion burden, irrespective of energy source. Thus, while PFA may reduce the risk of severe fibrotic remodelling, its application—especially in broader substrate modification—should be judicious, with continued monitoring of atrial function. Current evidence supports a cautious approach pending further long-term mechanistic data.

PFA’s impact on endothelial integrity and thrombogenicity remains an area of ongoing investigation. While it avoids thermal charring, the delivery of high-voltage electric fields may acutely disrupt the endocardium. Haemolysis observed in the *NEMESIS-PFA* study^34^ suggests intra-procedural blood element injury, raising concerns about transient prothrombotic risk prior to endothelial recovery. Although PV vein stenosis has not been reported—implying preservation of PV endothelial architecture—transient arterial wall changes seen in coronary vasculature post-PFA warrant further vascular safety evaluation,^44^ particularly near the atrial septum (proximity to aorta) or AV groove (proximity to coronary arteries). From a neuroanatomic standpoint, PFA’s relative sparing of ganglionated plexi compared to RFA may alter autonomic modulation. While this could reduce unintended vagal denervation, it also raises concerns about diminished efficacy in cases where targeting autonomic inputs contributes to arrhythmias. These mechanistic uncertainties highlight the need for further pre-clinical and longitudinal human studies.

In summary, PFA while biophysically distinct from thermal modalities warrants critical scrutiny as its clinical use expands. The assumption that non-thermal energy confers universal physiologic benefit remains unproven. Although acute preservation of tissue architecture has been observed, emerging evidence of endothelial disruption, haemolysis, and transient atrial dysfunction indicates that electroporation carries its own spectrum of biologic effects. Moreover, the implications of repetitive high-voltage exposure on myocardial, vascular, and autonomic substrates are not fully elucidated. Accordingly, broad adoption of PFA should be tempered by mechanistic vigilance and rigorous longitudinal assessment, rather than inferred safety from absence of thermal injury alone.

### Comparative Efficacy and Technical Heterogeneity of Pulse Field Ablation

While PFA offers distinct advantages—including myocardial selectivity and a favourable safety profile—current evidence does not support its universal replacement of thermal ablation. Randomized trials have shown comparable one-year efficacy for paroxysmal AF between PFA, RFA, and cryoballoon ablation (∼75–80%).^2,5,50^ Despite PFA’s rapid evolution, RFA and cryoablation benefit from decades of refinement. Even laser balloon ablation, which demonstrated similar efficacy to cryoablation,^51^ failed to gain widespread traction, underscoring that non-inferiority alone is insufficient to supplant established technologies.

Significant heterogeneity among PFA systems—including waveform shape, pulse parameters, and catheter design—limits cross-platform comparability. Monophasic pulses produce larger lesions but trigger intense skeletal muscle activation, requiring general anesthesia.^52^ In contrast, biphasic waveforms reduce neuromuscular activation and allow conscious sedation but demand higher energy for comparable efficacy.^52,53^ Pre-clinical data demonstrate that waveform frequency and polarity dramatically affect lesion size and safety: a 450 kHz biphasic pulse required ∼2000 V/cm for cell death vs. 600 V/cm for monophasic pulses.^52,54^

Catheter configuration further modulates lesion characteristics. Balloon and multi-electrode array designs concentrate energy over broader areas and enable efficient single-shot ablation, whereas focal catheters require sequential application but may allow greater precision.^55,56^ Energy efficiency varies widely; in silico models show that circular or balloon catheters deliver 10–12% of generator energy to tissue, while multi-electrode configurations transmit <3%.^57^ Such discrepancies translate into procedural variability: Farapulse’s biphasic pentaspline system demonstrated 96% durable PVI at three months and 84.5% arrhythmia-free survival at one-year,^58^ while CENTAURI’s focal PFA system (Galvanize Therapeutics) reported 84–92% PVI durability in optimized cohorts.^59^ Notably, silent cerebral lesion rates differ across devices, with reports of ∼10% for Medtronic’s Sphere-9^60^ and 18.5% for Farapulse’s Farawave catheter.^37^

Delivery architecture also confounds energy comparisons. ADVENT trial^5^ compared single-shot PFA systems to point-by-point RFA or cryoablation conflating catheter design with energy type. Such designs make it difficult to isolate the effect of pulsed-field energy from catheter architecture. Newer platforms break this paradigm. The CENTAURI system (Galvanize Therapeutics) delivers PF energy through standard contact-force RF catheters. In the prospective ECLIPSE AF study, Anić *et al*. reported that 100% of targeted PV were isolated acutely (322/322 veins) and that one-year freedom from atrial tachyarrhythmia was 80.2%.^61^ By contrast, Medtronic’s Sphere-9 lattice-tip catheter (capable of both RFA and PFA) was tested in a large randomized trial and demonstrated significantly shortened ablation and overall procedure times compared to conventional point-by-point RFA, while meeting non-inferiority criteria for 12-month arrhythmia-free survival.^31^ These data illustrate that both energy source and catheter design influence clinical outcomes.

In conclusion, no single ablation modality currently demonstrates superiority. While PFA offers distinct safety advantages—most notably, the near elimination of thermal collateral damage—these benefits are offset by emerging risks, including myocardial stunning, haemolysis, and coronary vasospasm. Importantly, most commercially available PFA platforms remain optimized primarily for PVI, with limited clinical validation in non-PV substrates. Moreover, the lack of standardization across waveforms, dosing protocols, and catheter architectures underscores that ‘PFA’ represents a heterogeneous group of technologies rather than a unified technique. Thus, cross-platform generalization is not ideal without dedicated validation studies. Until reproducible long-term data across AF subtypes emerge, lesion-specific energy selection remains the most rational strategy.

### Cost, training, and accessibility considerations

Beyond clinical performance, practical factors influence whether a technology can or should become the standard of care. An analysis in the UK reported median procedural costs for PFA about 12% higher than RFA and 23% higher than cryoablation, driven largely by the price of pulse field generators and catheters.^62^ In the United States, where device pricing differs, some estimate pulse field equipment costs to be three to four times higher than established platforms.^63^ While costs may come down with competition and scale, presently the financial barrier is non-trivial, especially for smaller centres. Hospitals need to budget for new capital equipment and disposables; one electrophysiology laboratory estimated roughly $2000 extra per PFA case compared to RFA or cryoablation.^64^ Until cost parity is reached, mandating PFA for all cases could strain healthcare resources or limit patient access in cost-constrained systems.

Transitioning to PFA presents significant training and workflow challenges, particularly for electrophysiologists accustomed to point-by-point RFA. The procedural mechanics of manipulating a large multi-electrode array resemble cryoablation techniques more than traditional RFA, necessitating retraining in single-shot catheter deployment and maneuvering. Moreover, PFA systems involve novel generator interfaces and require familiarity with pulse-specific parameters such as waveform configuration, voltage amplitude, and burst protocols—elements unfamiliar to many practitioners. Broad implementation will demand comprehensive education not only for operators but also for electrophysiology laboratory teams, encompassing protocol standardization and safety oversight. Resource-limited centres may find it impractical to sustain parallel competencies across multiple energy platforms and may opt to retain established technologies with well-understood workflows. Additionally, early experience emphasizes the importance of controlled catheter handling, as excessive force or poor technique with the relatively non-compliant PFA catheter may increase procedural risk, including perforation.^64^ These factors collectively challenge the narrative of PFA's operational simplicity and underscore the need for structured training pathways to ensure safe and effective integration into clinical practice.

Despite regulatory approval in both Europe and the United States as of 2025, the availability and clinical integration of PFA remain heterogeneous. Sedation needs have meaningful operational and geographic consequences for PFA adoption. Centres that rely primarily on conscious sedation—common in parts of Europe and in ambulatory or resource-limited environments—may face constraints implementing PFA systems that generate robust skeletal muscle capture and thus typically require general anaesthesia (GA) or deep sedation for safe delivery.^65^ In several countries, institutional policies prohibit electrophysiologists from administering deep sedation, necessitating anaesthesia team involvement, which may be unavailable or inconsistently staffed. This creates scheduling bottlenecks, increases procedural cost, and limits throughput, particularly in high-volume public systems.^66^ Conversely, centres with routine electrophysiology-dedicated anaesthesia support—more common in the United States—can integrate PFA without major workflow disruption, but at higher resource expenditure. These disparities underscore a practical reality: sedation infrastructure, staffing models, and local regulatory frameworks will directly influence which PFA technologies are viable, how widely and rapidly they can be adopted, and whether conscious-sedation–centric programmes can incorporate PFA without significant operational redesign.

These operational disparities underscore why it is pre-mature to position PFA as a universal standard of care. A more realistic trajectory is phased adoption, with early implementation concentrated in high-volume academic centres that can support GA-based workflows, rigorous monitoring, and procedural optimization. Broader dissemination should follow only as the technology’s efficacy, safety profile, durability, and cost-effectiveness are validated across diverse practice environments. Therefore, PFA should be viewed as an important and rapidly evolving addition to the ablation armamentarium rather than a wholesale replacement, and its optimal role within the therapeutic landscape will evolve as evidence and access expands.

### Gaps and future directions

While there is enormous promise of PFA several issues still remain to be addressed. 1) *Catheter optimization*: The current generation of single-shot catheters while providing some workflow efficiencies have significant biophysical limitations. Key design parameters influencing catheter performance include electrode geometry and surface area, inter-electrode spacing, polarity configuration (unipolar vs. bipolar), energy delivery architecture, irrigation strategy, and whether the catheter employs an open or closed design. 2) *Waveform optimization*: Optimization of PFA biophysics requires systematic evaluation of key parameters, including pulse amplitude, pulse width, number of pulses, and inter-pulse interval. These factors significantly influence lesion formation, safety, and efficacy, and must be empirically validated for each specific catheter configuration. 3) *Clinical workflow optimization*: Considerable procedural variability exists in the clinical application of PFA, including lesion stacking, total pulse deliveries, catheter repositioning, tissue contact, and adjunctive use of intra-cardiac echocardiography or electroanatomic mapping. These factors introduce complexity and may significantly influence lesion durability and safety. Compared to the relatively standardized biophysics of single-tip RFA, PFA involves a more intricate set of variables, underscoring the need for rigorous, scenario-specific validation. Regulatory frameworks must account for this complexity in evaluating device performance. Future system designs may benefit from more streamlined, lower-profile catheters with dual-energy capabilities, allowing operators to switch between PFA and RFA to tailor lesion delivery based on anatomical and clinical demands.

In conclusion, PFA represents a meaningful advance in AF ablation, offering procedural efficiency and a favourable acute safety profile, particularly with regard to avoiding thermal injuries. Early randomized trials and registries demonstrate non-inferior efficacy compared to RFA and cryoablation in paroxysmal AF. However, these findings must be interpreted within the context of evolving evidence. PFA's performance in persistent and long-standing persistent AF remains uncertain, and concerns about lesion durability, myocardial and vascular effects, and procedural variability underscore the need for continued scrutiny. Furthermore, system-level factors—training requirements, cost, and limited device availability—pose practical barriers to universal adoption. While enthusiasm for PFA is understandable, its integration should be grounded in rigorous evidence and clinical judgement. Current data support its selective use, particularly in anatomically high-risk cases or where procedural time is a priority, but not as a blanket replacement for established thermal modalities. Until longer-term safety and efficacy across diverse patient populations are demonstrated, a balanced, patient-specific energy selection strategy remains essential.
